# Supplementary material for: Integrated 3D printing of flexible electroluminescent devices and soft robots
Source: Nat Commun. 2022 Aug 23;13:4775. doi: 10.1038/s41467-022-32126-1 (PMC9399151; doi:10.1038/s41467-022-32126-1)
Supplement: Supplementary file 1 — Supplementary Information [file 41467_2022_32126_MOESM1_ESM.pdf]

Supplementary Information: Integrated 3D Printing of Flexible Electroluminescent Devices and Soft Robots

Pei Zhang,[1] Iek Man Lei,[1] Guangda Chen,[1] Jingsen Lin,[1] Xingmei Chen,[1] Jiajun Zhang,[1] Chengcheng Cai,[1] Xiangyu Liang,[1] Ji Liu[1,2,3**]

E-mail: liuj9@sustech.edu.cn

[1] Department of Mechanical and Energy Engineering, Southern University of Science and Technology, Shenzhen 518055, China.

[2] Shenzhen Key Laboratory of Biomimetic Robotics and Intelligent Systems, Department of Mechanical and Energy Engineering, Southern University of Science and Technology, Shenzhen, 518055, China.

[3] Guangdong Provincial Key Laboratory of Human-Augmentation and Rehabilitation Robotics in Universities, Southern University of Science and Technology, Shenzhen, 518055, China.

S.I. Methods and Synthesis

Materials and Chemicals.

Unless otherwise specified, the materials and chemicals used in this work were used directly without further purification. Polyacrylic acid (PAA, average M_w = 450,000 Da), zwitterionic monomer 3-dimethyl (methacryloyloxyethyl) ammonium propane sulfonate (DMAPS) and poly(ethylene glycol) diacrylate (PEGDA, M_w = 400 Da) were purchased from Macklin. The ionic liquid molecule of 1-ethyl-3-methylimidazolium ethyl sulfate (EMES) was obtained from Adamas. Fluoroelastomer poly(vinylidene fluoride-*co*-hexafluoropropene) (PVDF-HFP, Daiel G801) was kindly provided by Daikin. ^{1}H,^{1}H,^{2}H,^{2}H-perfluorooctyl acrylate (TEAc) was purchased from Shang Fluro *Co. Ltd.* Commercially available ZnS phosphor particles were purchased from Shanghai Keyan Phosphor Technology. Photo-initiators, 2-hydroxy-4'-(2-hydroxyethoxy)-2-methylpropiophenone (Igacure 2959) and diphenyl-(2,4,6-trimethylbenzoyl)-phosphine oxide (TPO), were purchased from TCI and Adamas, respectively. Fume silica (AEROSIL 200) was kindly provided by King Chemical *Co. Ltd.*

S.II. Instrumentation and Measurements

Rheological Characterization.

Rheological measurement was conducted using a rotational rheometer (DHR rheometer TA instruments) with a parallel plate (20 mm in diameter, 200 μm in gap). Shear viscosity of the inks was measured in a steady-state flow mode over a shear rate ranging from 10^{-2} to 10^3 s^{-1}, and the storage (G') and loss moduli (G'') as a function of shear stress were measured. The yield stress was taken at the point where $G' = G''$. Dynamic oscillatory amplitude sweep was conducted in the amplitude range from 1% to 1000%, at a constant frequency of 10 rad s^{-1}. Real-time photorheological measurements were done on the DHR Rheometer equipped with a UV accessory that exploited a light guide and reflecting mirror assembly to transfer

UV radiation from a high-pressure mercury light source. A UV light source (Exfo Omnicure S2000, wavelengths of 365 nm) with a triggering cable was attached to the DHR Rheometer. Once the UV radiation was on, time-dependent evolution of the mechanical properties (G' and G'') was measured at a constant amplitude of 0.5% and frequency of 10 rad s^{-1}. All the measurements were carried out at room temperature, and all results were processed using the TA Instruments *TRIOS* software.

Mechanical Testing.

The mechanical properties were measured using a Cellscale's Ustretch tensile machine, which was equipped with a 44-N load cell. Dog-bone samples with a dimension of 5 mm in width, 10 mm in gauge length, and 0.6 mm in thickness were stretched at room temperature. 180-degree peeling tests were carried out to quantify the interfacial toughness of joints between the 3D printed layers of different inks. The 3D printed samples were fabricated with a dimension of 50 (L) $\times$ 10 (W) mm. Tearing tests were also conducted to quantify the toughness of the ICE, ELE and IDE samples. The samples were fabricated with a dimension of 50 (L) $\times$ 10 (W) mm. All the tests were carried out at a deformation rate of 20 mm min^{-1}.

Characterisation of the Electrical Properties of the EL units.

Resistance of the ICE samples was measured with a TH2832 LCR meter. To track the change in the resistance upon mechanical deformation, the samples were connected to the LCR meter using conductive Ag pastes prior to unidirectional stretching.

Dielectric frequency spectra of the insulating dielectric elastomer (IDE) layer and the PDMS dielectric polymers were measured using a LCR meter (E4980A) with a DMS-2000 dielectric temperature spectrum system. In order to prepare the double-sided electrode films for the measurement, the samples with a dimension of 10 mm (L) $\times$ 10 mm (W) $\times$ 0.5 mm (t) were laminated with PET masks that had a 6 mm diameter punched-through hole. A thin film of platinum was then deposited over the sample using ion sputtering (Mciooo

ion sputter, HITACHI). The permittivity measurements of the samples were performed at a frequency range from 20 to 10^6 Hz at room temperature.

Imaging and Image Analysis.

Photos and videos in this work were taken with a MILC SONY camera (α7R4, Japan). Green dye (fluorescein) was used to stain the 3D printed samples for better visualization effect. Optical microscope (Mshot MD30) was used to assess the micro-structural features of the 3D printed patterns. Inverted fluorescence microscope (Olympus IX73) was used to evaluate the fidelity of the 3D printed ELE filaments and the conformity of the interfaces between different layers of inks. The blue luminescence of the ELE ink was resulted under an excitation wavelength λ_{ex} of 530 nm. The ICE layers were stained by Nile Red (0.1 *wt.*%) with an excitation wavelength λ_{ex} of 530 nm, and an emission wavelength λ_{em} of 570 nm. The morphologies of the 3D printed multi-layer interface were also assessed using a field emission scanning electron microscopy (Merlin, Zeiss) at a current of 100 pA and an acceleration voltage of 5 kV. Prior to SEM observation, the 3DP multi-layered samples (*ca.* 11 layers, *ca.* 2.5 mm in thickness) were sliced at a thickness of 200 μm using a freezing microtome (Leica CM1950 Cryostat).

Luminance Quantification.

The luminance and chromaticity of the 3D printed EL devices were measured with an integrating sphere (FOIS-1) and an QE Pro spectrometer, respectively. A high-voltage AC power supply (6705 linear programmable AC power source, ECC industries), coupled with voltage amplifier (OPT.624, output voltage 600 V) and frequency amplifier (OPT. 625, output frequency 1 kHz), was employed to drive the electroluminescent devices. Optoelectronic characteristics of the EL devices were quantified using a QE Pro spectrometer by gradually increasing the voltage from 50 V to 600 V. The amplifier provides monitoring terminals output voltages (1 V/1 kV) and current (1 V/4 mA), which were recorded with a Keysight

DSOX2024A oscilloscope.

Finite Element Analysis (FEA) Simulation.

A commercial software (ABAQUS 2018, SIMULIA) was used to study the stress and strain distributions within the 3DP samples and the physically-laminated samples (control) when a 35% strain was applied to the samples. The sample, which consists of ICE and ELE layer, has a dimension of 150 mm (length) × 10 mm (width) × 2.2 mm (height). The simulation was performed using the elastic-plastic constitutive calculation model in ABAQUS. The interfacial toughness between the ICE and ELE layers of the 3DP sample was set as 150 J m^{-2} in the simulation, whereas the friction coefficient between layers of the physically-laminated sample was set as 0.45.

S.III. Supplementary Results.

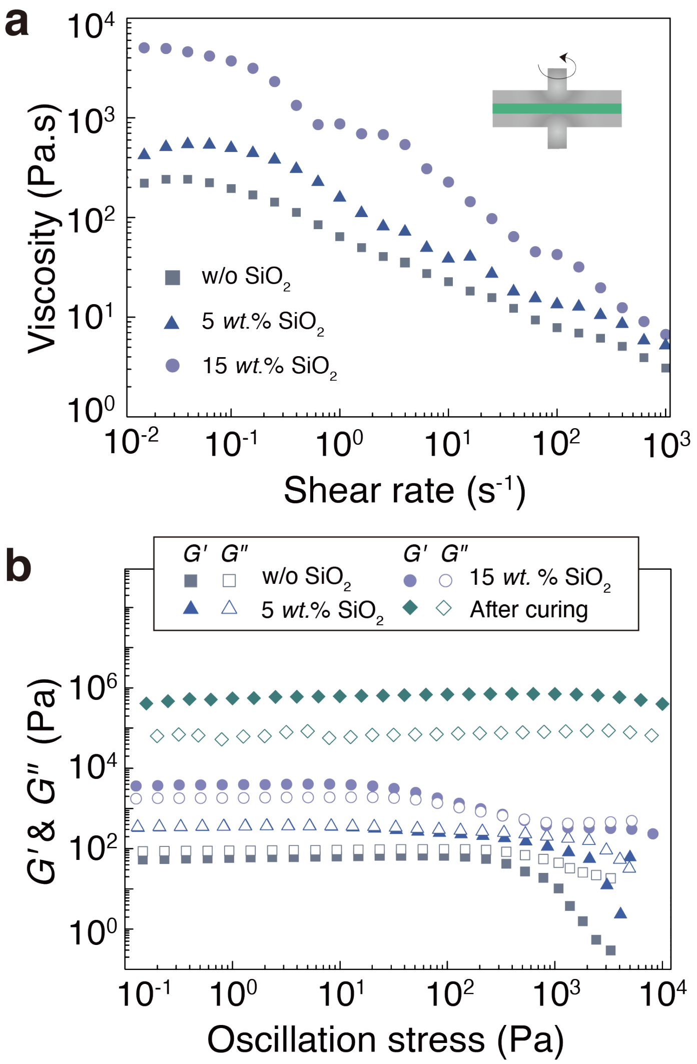

Supplementary Figure 1: **Rheological properties of the ion conducting elastomer (ICE) inks. (a).** Plotting of apparent viscosity of the ICE inks of various compositions as a function of the shear rate of the ICE inks at 25 °C. **(b).** *G*' and *G*" of the different formulations of the ICE inks measured from dynamic room-temperature oscillation sweeping with the oscillation stress ranging from $10^{-1} \sim 10^{4}$ Pa. The chemically-crosslinked elastic network ('after curing' samples) was obtained by curing the ICE inks under UV radiation.

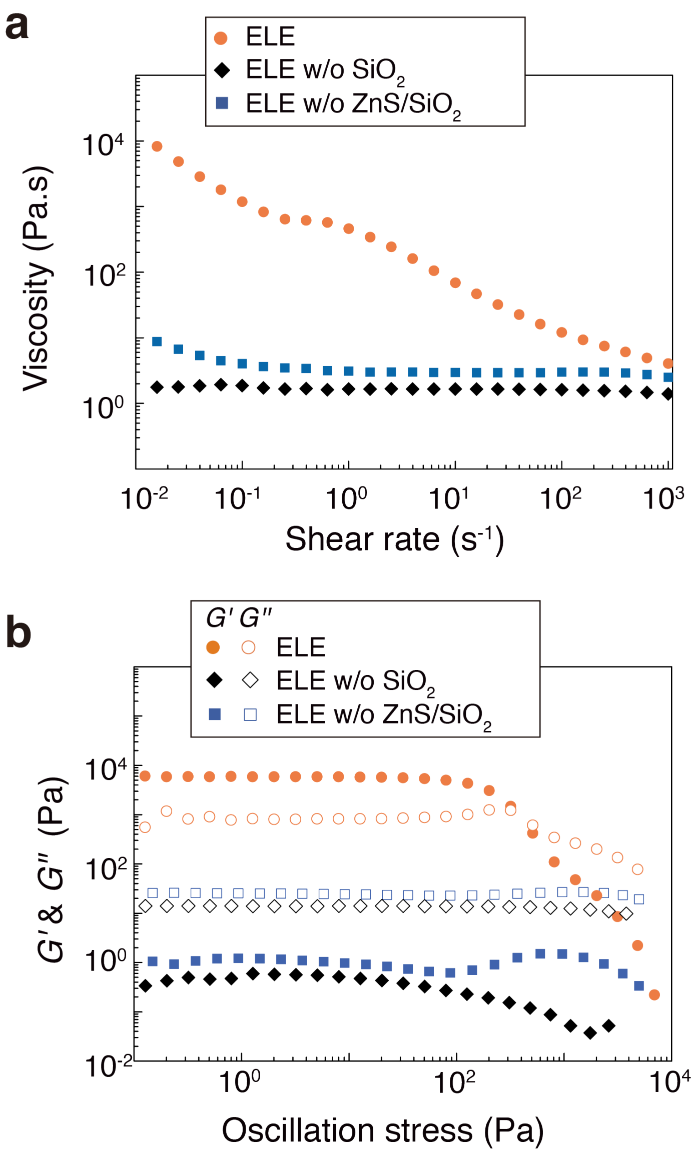

Supplementary Figure 2: **Rheological properties of the electroluminescent elastomer (ELE) inks. (a).** Plot of the apparent viscosity of ELE inks with or without SiO_2 and ZnS against the shear rate at 25 °C. **(b).** G' and G" values of the the ELE inks without ZnS and/or SiO_2 nanofillers measured from dynamic room-temperature oscillation sweeping with the oscillation stress ranging from 10^{-1} to 10^4 Pa. A slight change in the rheological properties was occurred when ZnS particles were added, and no yielding occurred for the ELE ink without SiO_2 and the ELE ink without ZnS/SiO_2. Viscoelastic inks were obtained when silica nanofillers were added (ELE ink), featuring distinct stress yielding which were highly desirable for DIW.

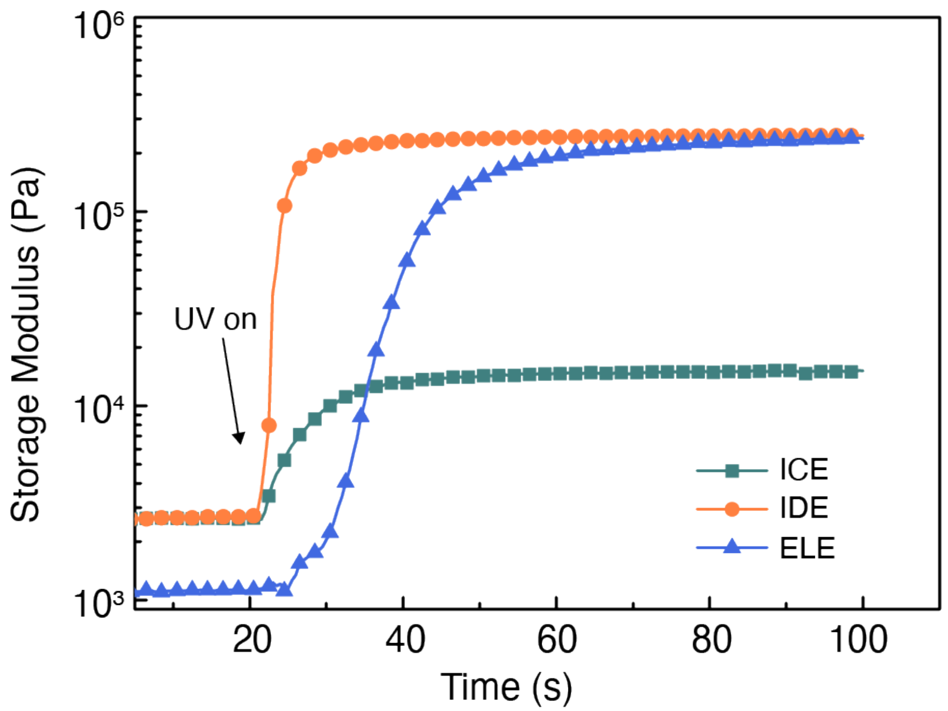

Supplementary Figure 3: **Gelation kinetics of the functional inks.** Representative plot of the storage moduli of the ICE, IDE and ELE inks against the UV radiation exposure time (420 nm, 20 mW cm^{-2}).

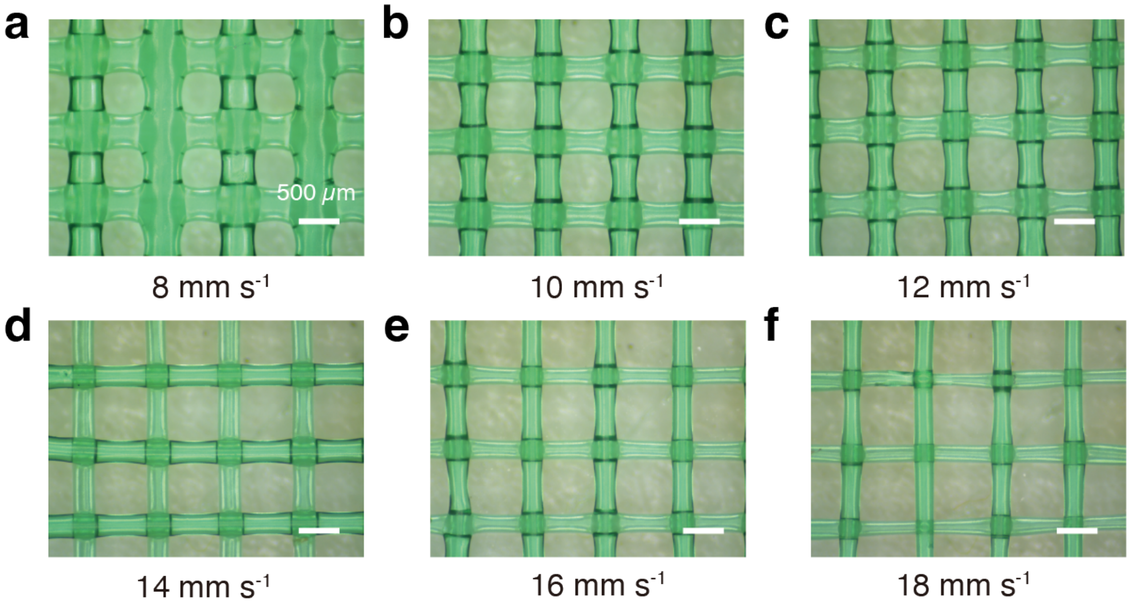

Supplementary Figure 4: **The print resolution of the ICE inks at various printing speed. (a-f)** Optical microscopic images of the 3D printed grids of the ICE inks (PAA:DMAPS:EMES = 1: 5: 5 *mol.*, SiO_2 solid fraction of 15 *wt.*%) printed at various speeds (applied pressure was set at 300 kPa). Scale bar: 500 μm.

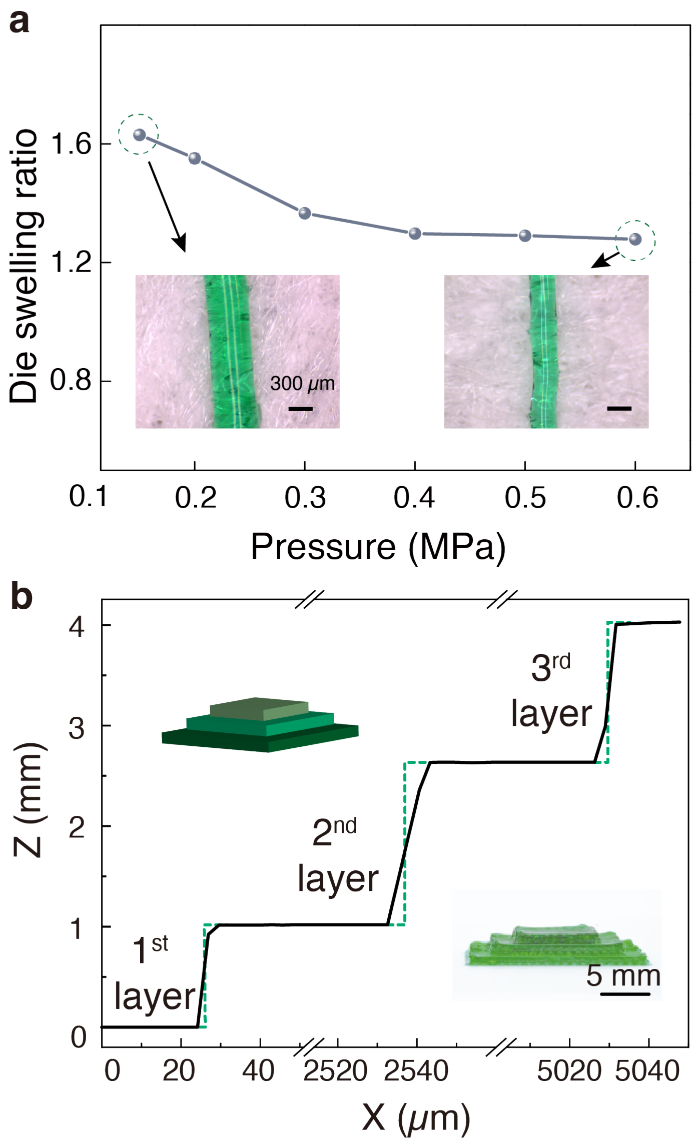

Supplementary Figure 5: **The print resolution of the ICE inks.** **(a)**. Plotting of the die swelling ratio against the applied pressure during 3D printing of the ICE inks. The die swelling ratio is defined as the ratio of the diameter of the 3D printed filaments to the diameter of the nozzle used. Insets are image of the 3D printed filaments obtained at the injection pressure of 0.12 MPa and 0.6 MPa, respectively. **(b)**. Variation in height across the length of the 3D printed staircase sample made of the ICE ink (solid black line) measured using laser confocal microscope (LCM), in comparison to the dimension of the CAD model (dashed green line). The staircase object had a dimension of 4 mm (H) $\times$ 15 mm (L) $\times$ 15 mm (W). The inset at the bottom right shows the photo of the 3D printed sample, and the inset at the upper left shows the design of the CAD model. The inks were stained with a green dye for better visualization. Data in **a** are presented as mean $\pm$ S.D., $n = 3$ independent samples.

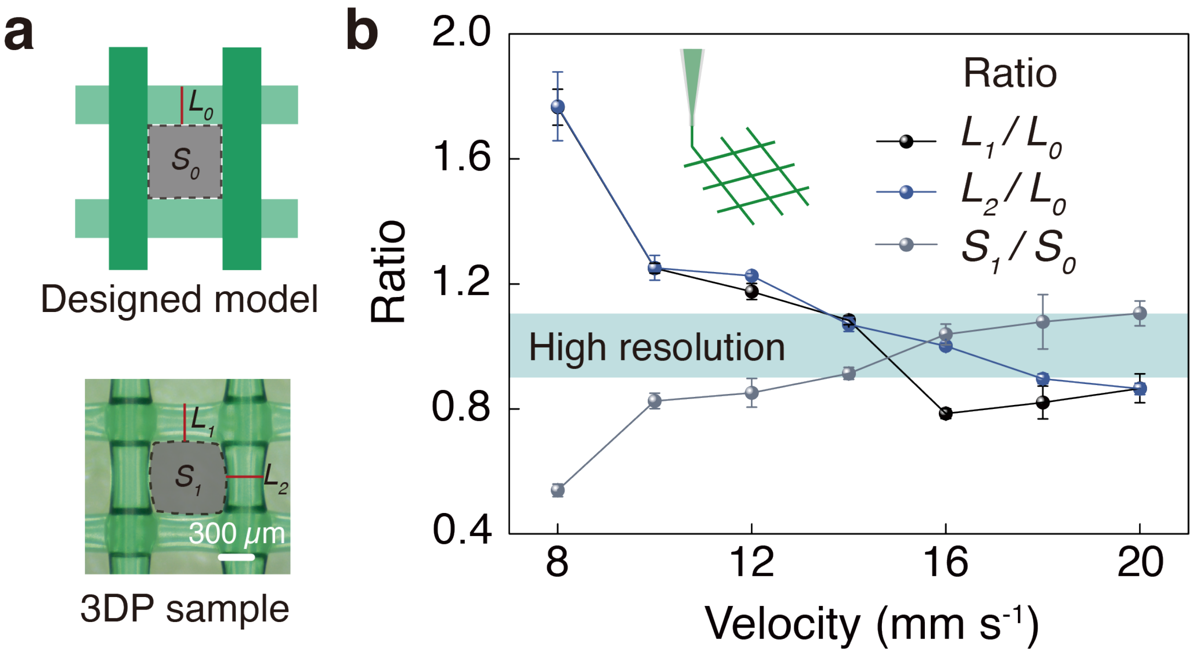

Supplementary Figure 6: **3D printing resolution of the ICE inks. (a).** Pictures illustrating the parameters used for evaluating the printing resolution of the ink, where L_0 and S_0 are the filament width and the internal space area between walls of the designed model, and L_1, L_2 and S_1 are the parameters of the 3DP samples. **(b).** Printability ratio (L_1/L_0, L_2/L_0 and S_1/S_0) of the ICE inks is plotted against the printing velocity with the applied pressure fixed at 0.3 MPa. Data in **b** are presented as mean ± S.D., $n = 3$ independent samples. Scale bar: 300 μm.

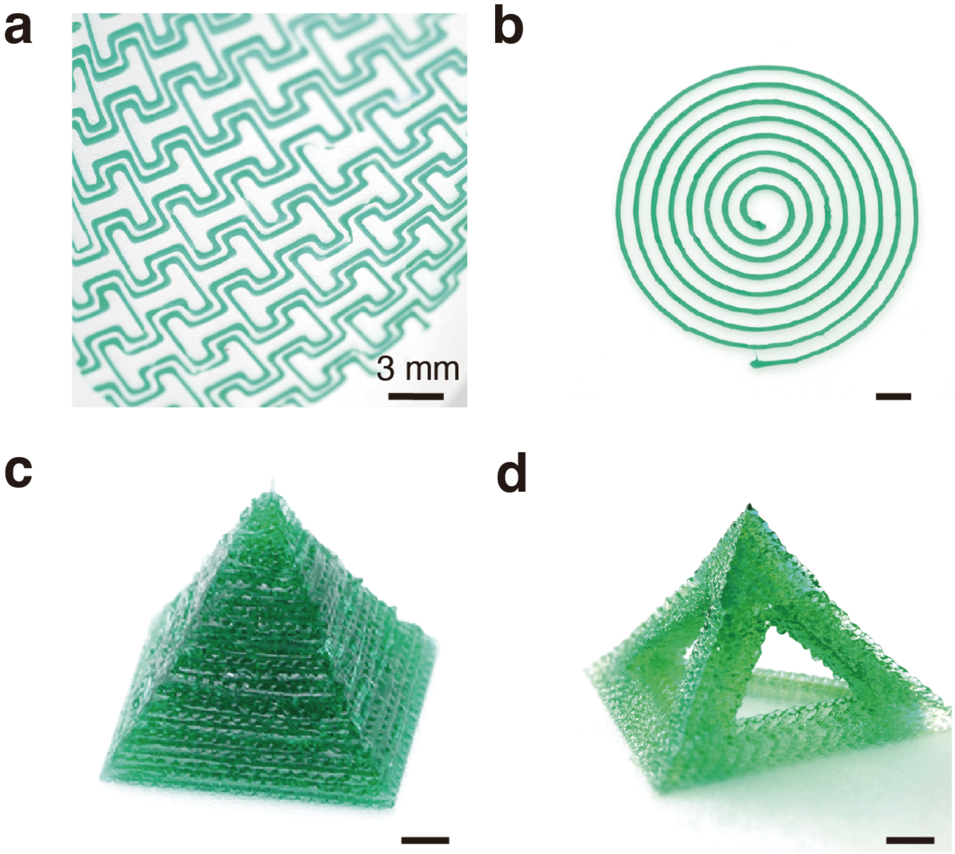

Supplementary Figure 7: **Design and 3D printing of ICE inks.** 3D printed complex structures using the ICE inks: maze circuit (**a**), spiral ring (**b**), solid pyramid (**c**) and 3D hollow pyramid (**d**). Scale bar: 3 mm.

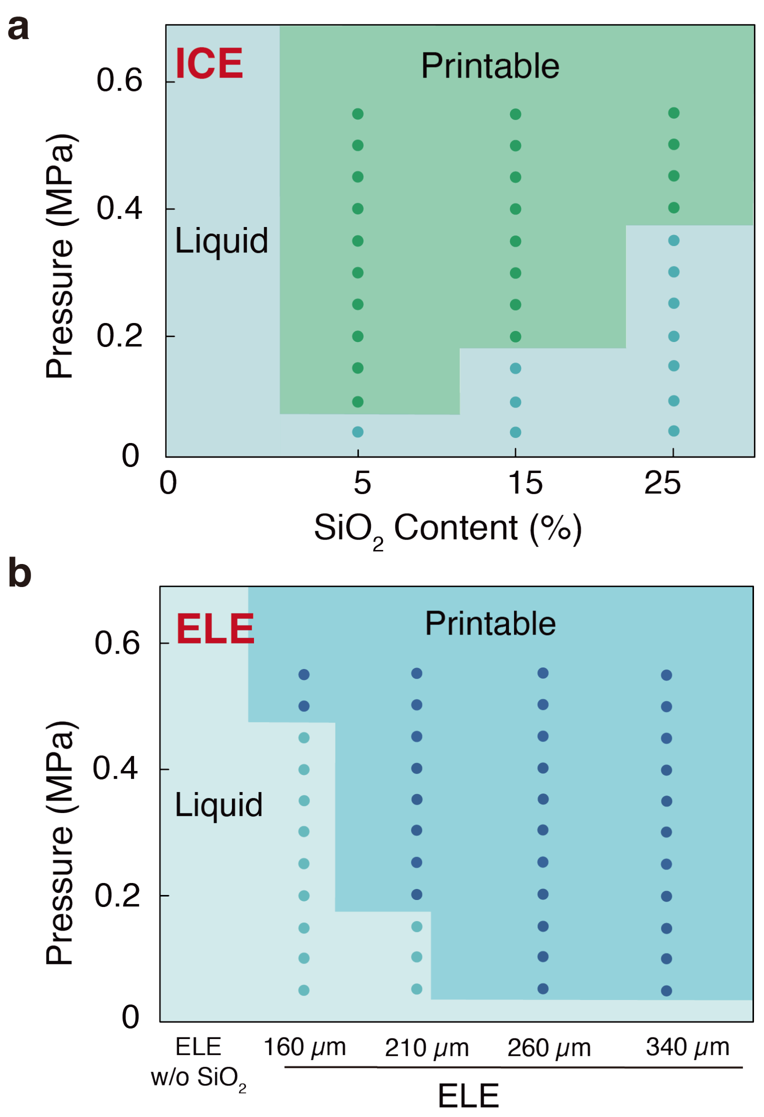

Supplementary Figure 8: **Printability of the ICE and ELE inks. (a).** Phase diagram illustrating the regions of SiO_2 content and applied pressure for a printable ICE inks. The ink can be printed into structures when the SiO_2 concentration ranged between 5 and 25 *wt.*% and the pressure was above a certain threshold as indicated in green in the graph. When SiO_2 concentration was below 5 *wt.*% or above 25 *wt.*%, the ink cannot be printed due to liquid spreading or particle clogging, respectively; and when the printing pressure was below the threshold value, the ink cannot flow out from the nozzle. **(b).** Phase diagram illustrating the printable region of the ELE inks. Without the addition of SiO_2 particles, the dielectric inks with or without ZnS particles flowed readily from the nozzle due to liquid spreading, while the addition of SiO_2 particles effectively improved the printability. The inks were printable at various nozzle diameters (*i.e.* 160, 210, 260 and 340 μm).

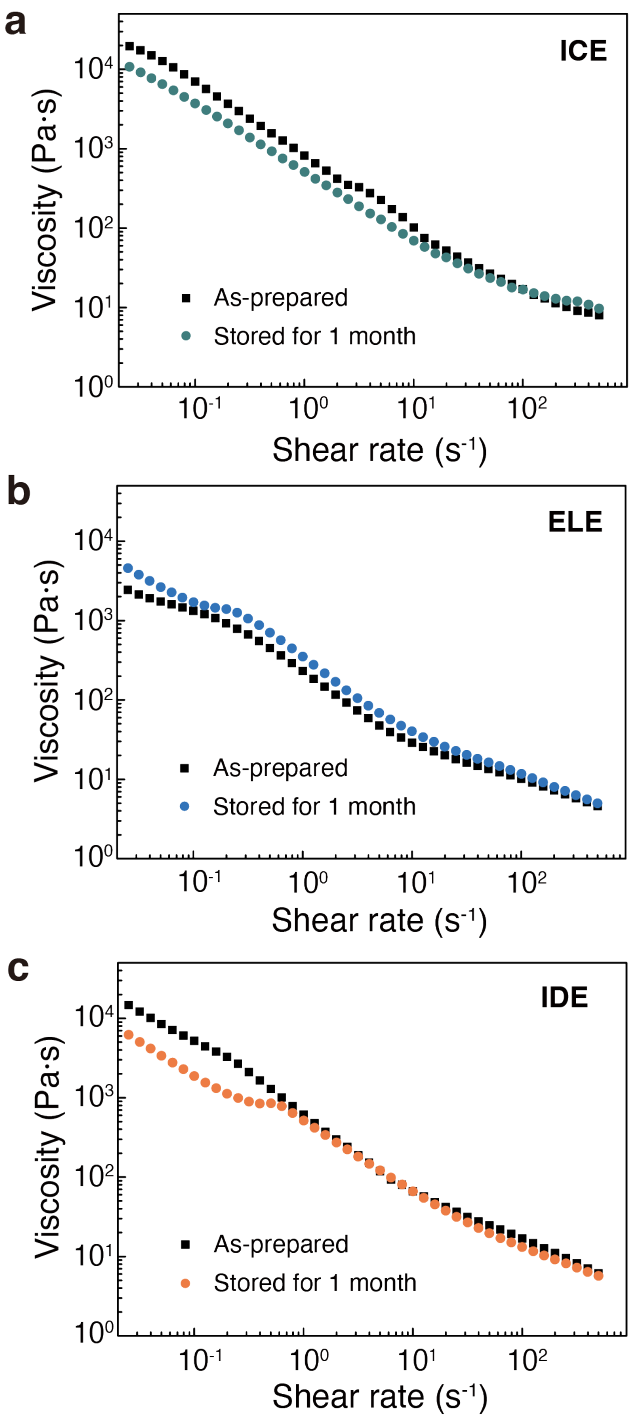

Supplementary Figure 9: **Stability of the inks during storage at -4 °C.** Representative rheological profiles of the three inks (**a**. ICE, **b**. ELE and **c**. IDE inks) upon storage in dark at -4 °C over one month. Nearly no change in the rheological properties was observed, indicating a good printability of the inks over long-term storage.

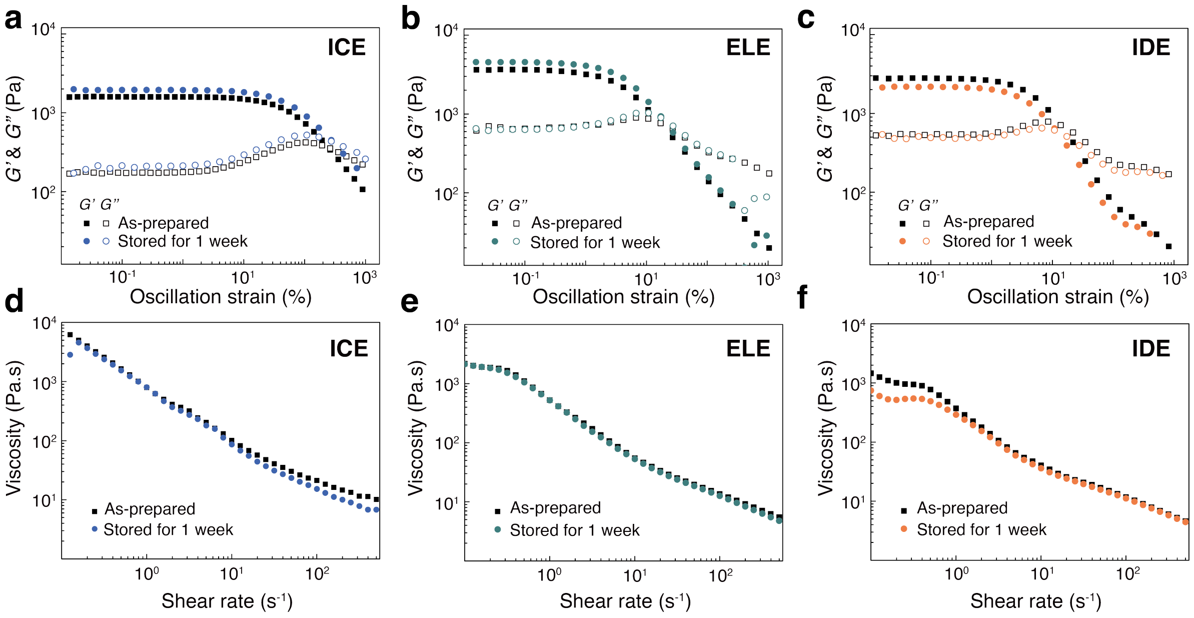

Supplementary Figure 10: **Stability of the inks during storage at room temperature.** Representative rheological storage and viscous modulus profiles (**a-c**) and the viscosity profiles (**d-f**) of the three inks (ICE, ELE and IDE inks) upon storage in dark at room temperature (25 °C) over one week. Nearly no change in the rheological properties was observed, indicating a good printability of the inks over long-term storage.

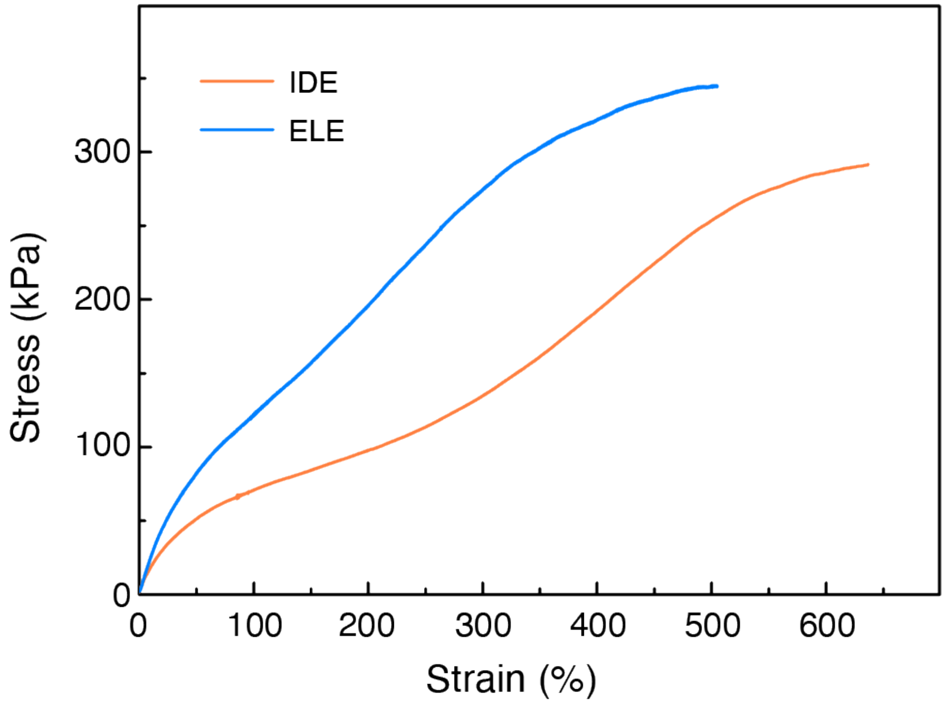

Supplementary Figure 11: **Mechanical properties of the electroluminescent elastomer (ELE) and insulating dielectric elastomer (IDE) samples.** Representative stress-strain curves of the electroluminescent elastomer (ELE) and the insulating dielectric elastomer (IDE) samples. The ELE ink is consisted of IDE, ZnS particles and silica nanofillers.

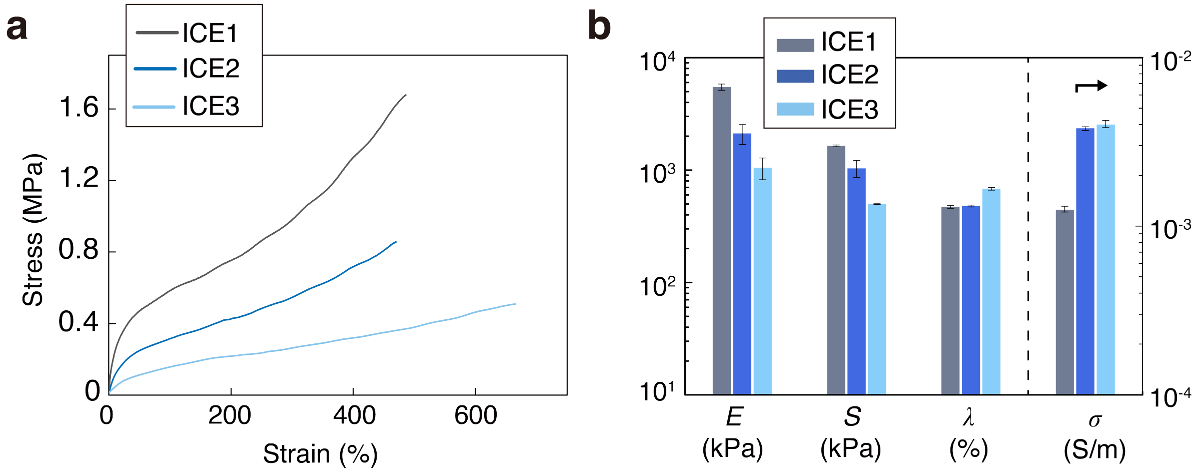

Supplementary Figure 12: **Mechanical properties of the ion conducting elastomer (ICE) samples. (a)**. Representative stress-strain curves of the ICE samples at various PAA/PDMAPS molar ratios. **(b)**. Summary of the physical parameters, including Young's modulus (E), fracture stress (S), elongation at break (λ) and ionic conductivity (σ) of the ICE layers at various PAA/PDMAPS molar ratios. Data are presented as mean ± S.D., n = 3 independent samples.

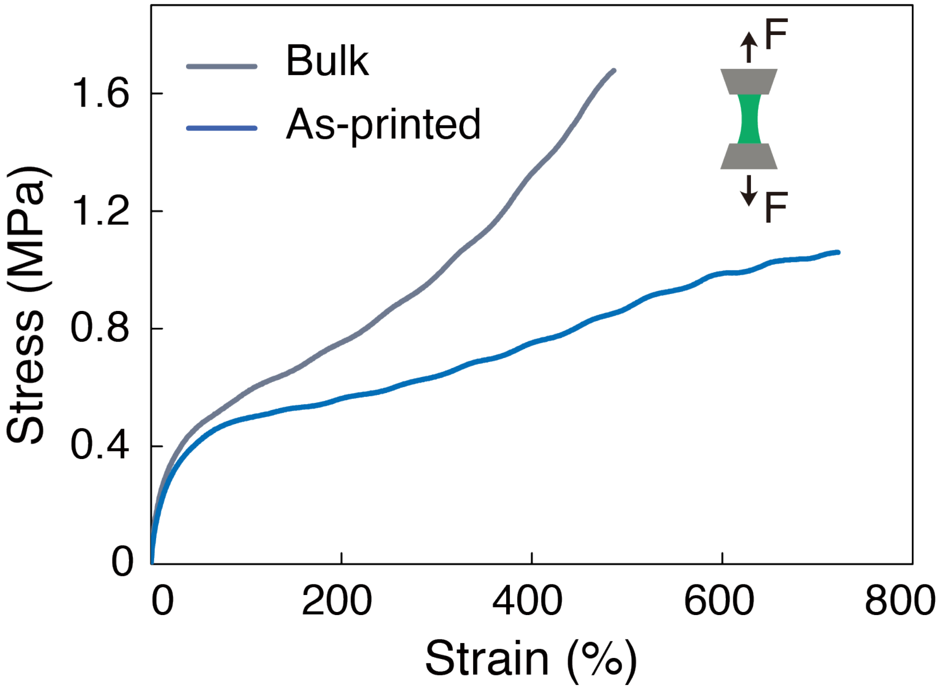

Supplementary Figure 13: **Mechanical behaviors of the 3DP ICE samples.** Representative stress-strain curves of the 3D printed ICE samples (As-printed, ink formulation: PAA:DMAPS:EMES = 1: 5: 5 *mol.*, and 15 *wt.*% SiO_2), while ICE samples of the same composition from direct molding (Bulk) were measured for comparison.

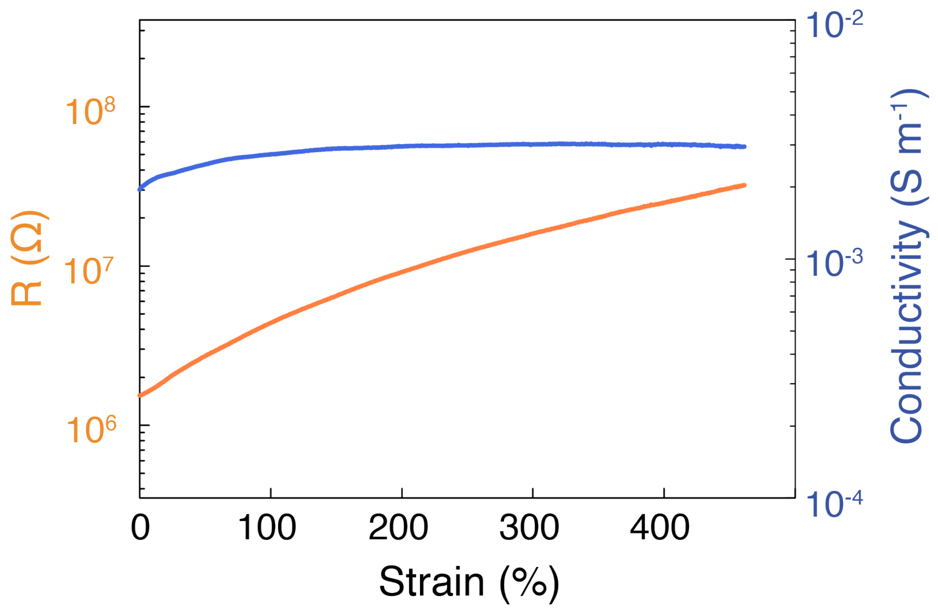

Supplementary Figure 14: **Evolution of the electrical properties of the ICE samples upon deformation.** Evolution of the resistance (R) and the ionic conductivity (σ) of the ICE samples during stretching at the strain range of $0 \sim 470\%$.

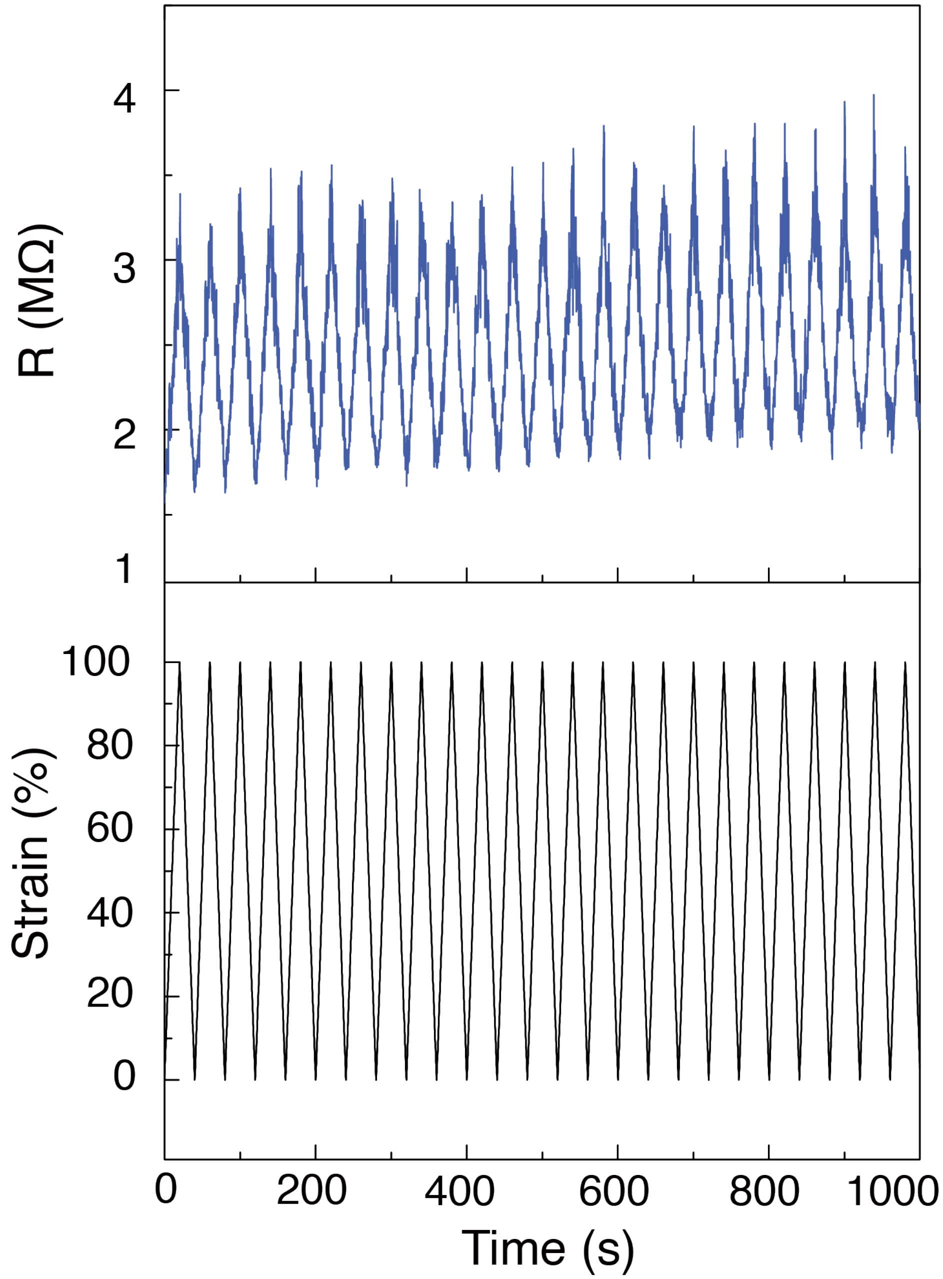

Supplementary Figure 15: **Robustness of the electrical properties of the ICE samples during multiple cyclic deformation.** Evolution of the resistance (R) of the ICE samples during cyclic deformation for 1000 s (deformation rate of 5% per second, 25 cycles in 1000 s), at a designated maximal strain of 100%.

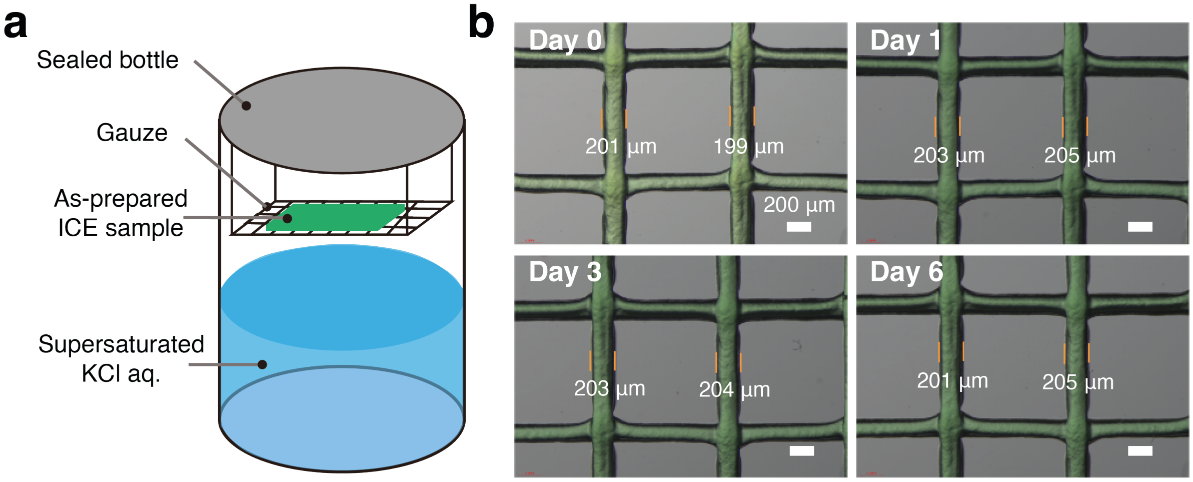

Supplementary Figure 16: **Structural stability of the 3D printed ICE filament under high-humidity condition. (a)**. Schematic showing the experimental setup for evaluating the dimensional change of the 3D printed filaments under high-humidity condition. **(b)**. Evolution of the dimension of the 3DP ICE filaments during the exposure to high-humidity environment at Day 0, Day 1, Day 3 and Day 6.

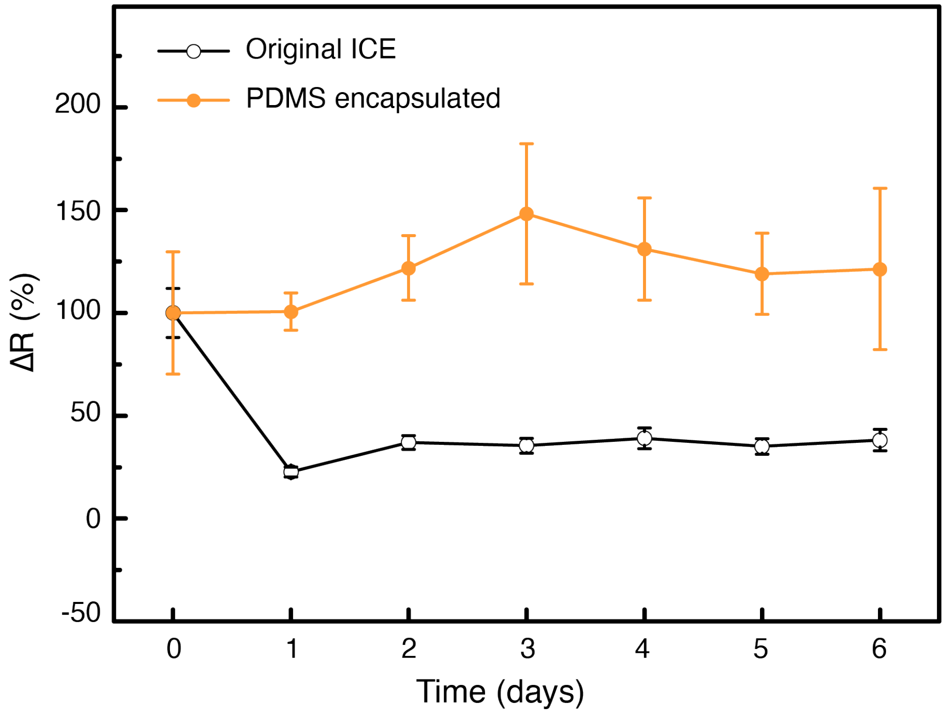

Supplementary Figure 17: **Change in the conductivity of the 3D printed ICE filament under high-humidity condition.** Evolution of the conductivity of the ICE samples and the ICE samples encapsulated in PDMS films for 6 days. Data are presented as mean $\pm$ S.D., $n = 3$ independent samples.

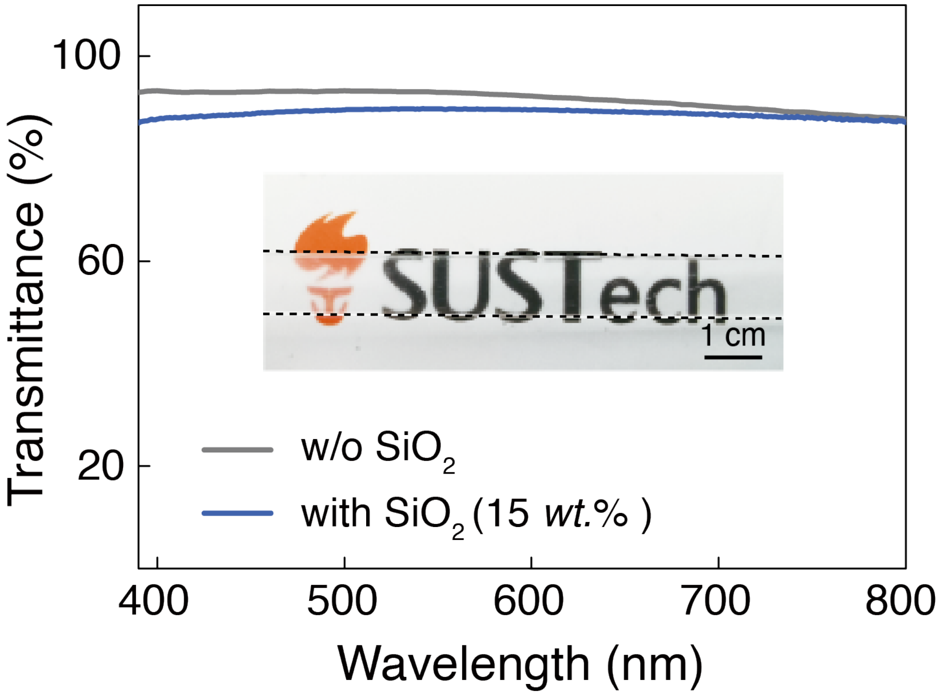

Supplementary Figure 18: **Transparency of the 3DP ICE samples.** Transmittance of the ICE samples with or without silica NPs fillers in the visible light range (390 nm $\sim$ 800 nm). Inset is the image of the 3DP ICE samples on a SUSTech logo, demonstrating the extraordinary transparency. The dot lines indicate the shape of the sample (scale bar: 1 cm).

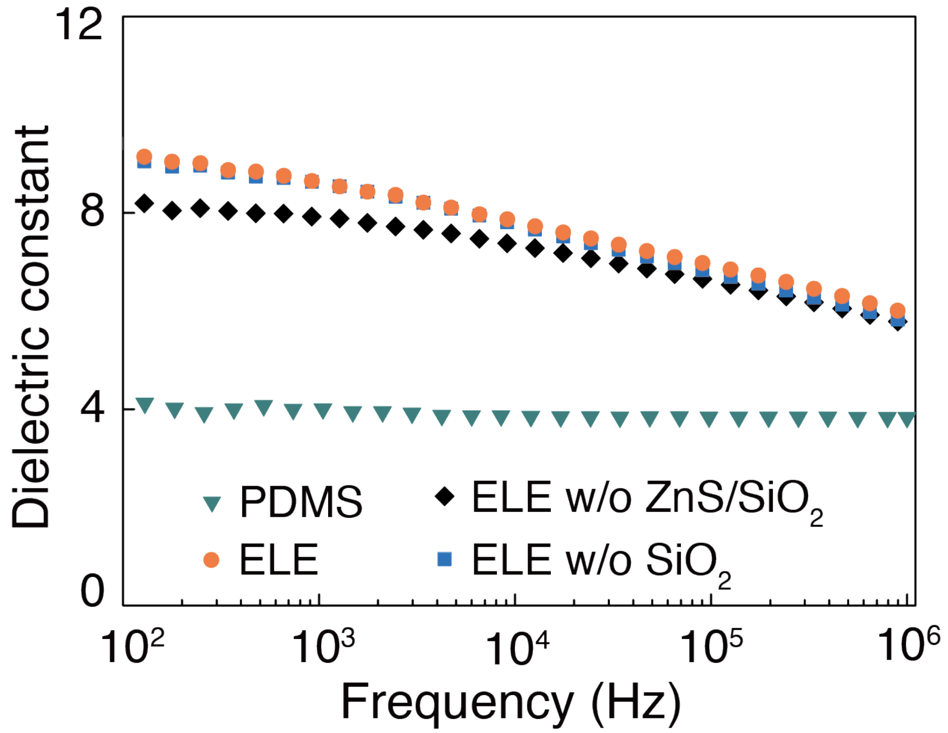

Supplementary Figure 19: **Dielectric properties of the ELE and IDE samples.** The dielectric constant of the dielectric elastomer (ELE, ELE w/o SiO_2, ELE w/o SiO_2/ZnS) samples at the frequency range of $10^2 \sim 10^6$ Hz, in comparison to the dielectric constant of pure PDMS (Ecoflex).

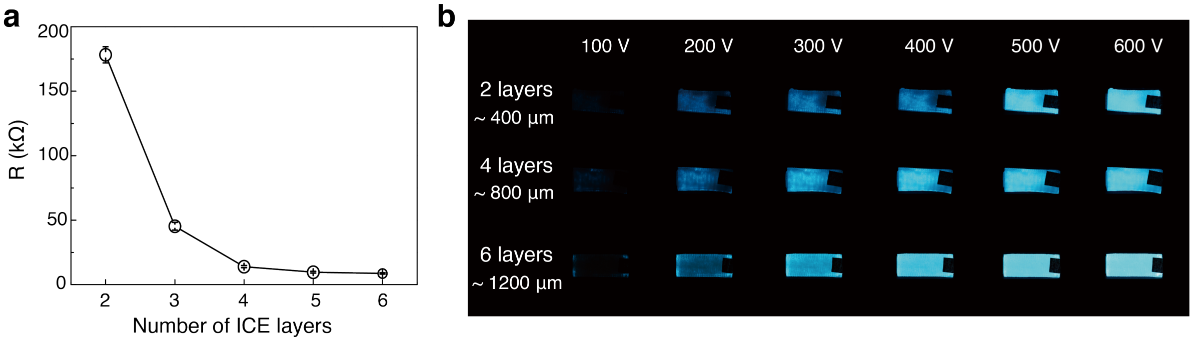

Supplementary Figure 20: **Electroluminescent performance of EL devices. (a)**. Resistance of the printed ICE samples with different number of layers. Each layer is ~ 200 μm. Data are presented as mean ± S.D., $n = 3$ independent samples. **(b)**. The effect of the ICE layer thickness (controlled by the number of layers) and the applied voltage on the electroluminescence intensity of the EL devices.

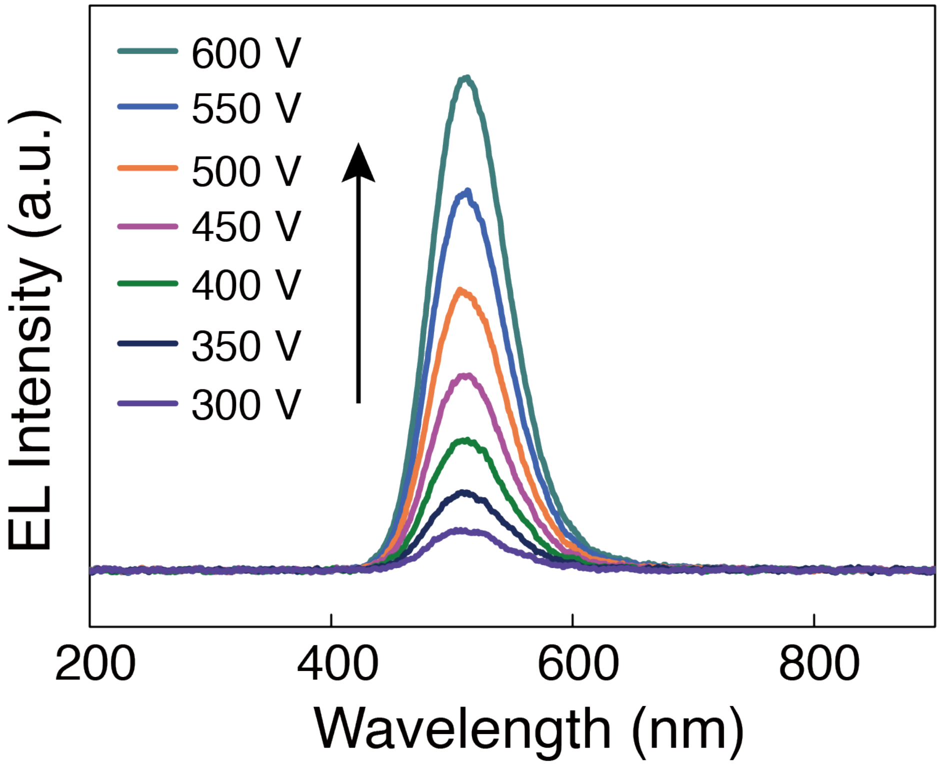

Supplementary Figure 21: **Electroluminescent emission of the ELE samples.** Electroluminescent emission spectra of the ELE samples (chemical formulation of IDE/ZnS(Blue)) activated at various voltages of the AC electric field.

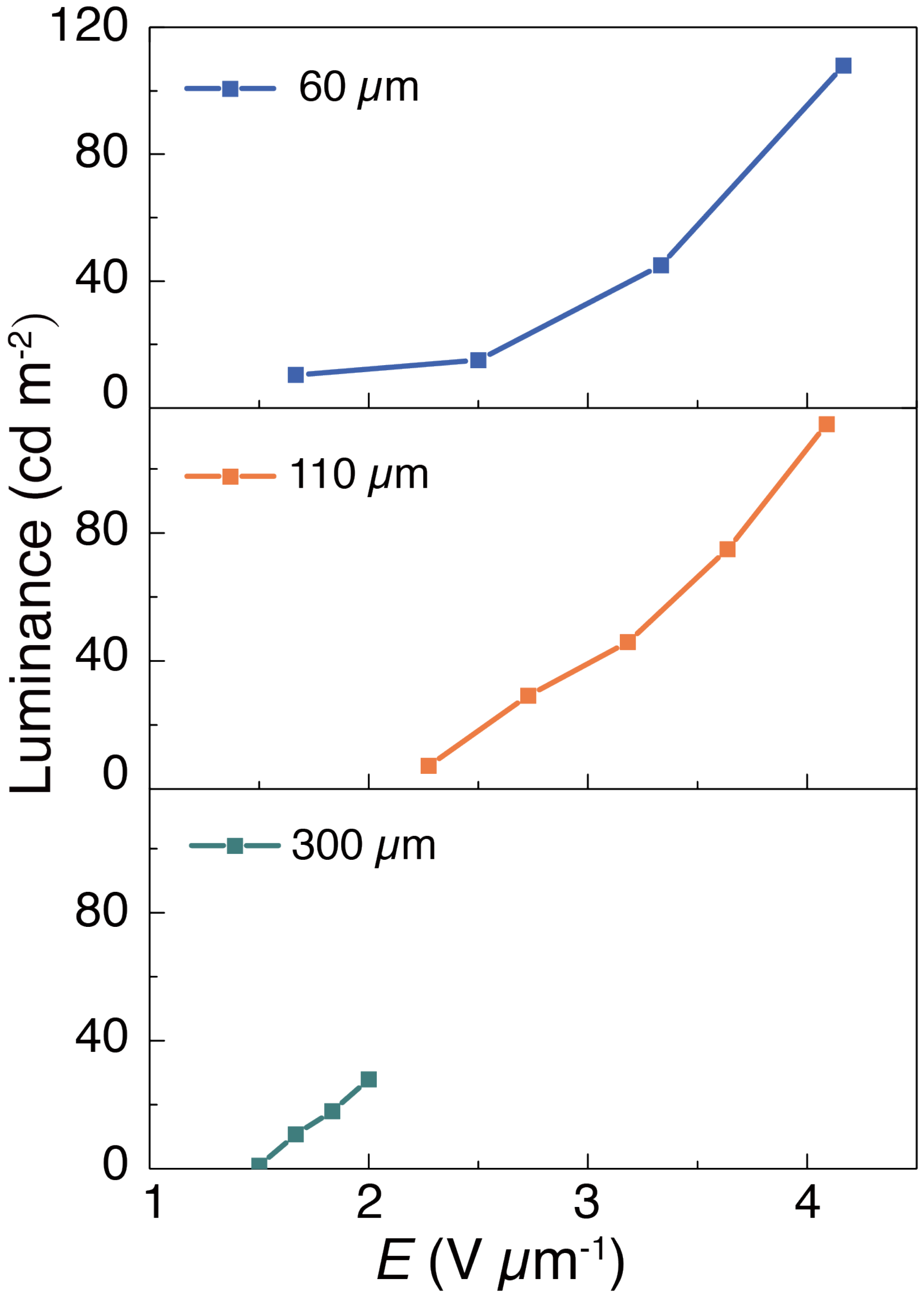

Supplementary Figure 22: **Effect of the sample thickness and the applied voltage per thickness on the electroluminescent emission of the ELE samples.** Plotting of the luminance intensity of the ELE samples with sample thickness of 60 μm, 110 μm and 300 μm against the applied voltage per thickness (V/μm). Frequency of the AC electric field was set as 1000 Hz. Data are presented as mean ± S.D., $n = 3$ independent samples. Due to slight difference among three repeated measurements, the S.D. of each data point is too small to see.

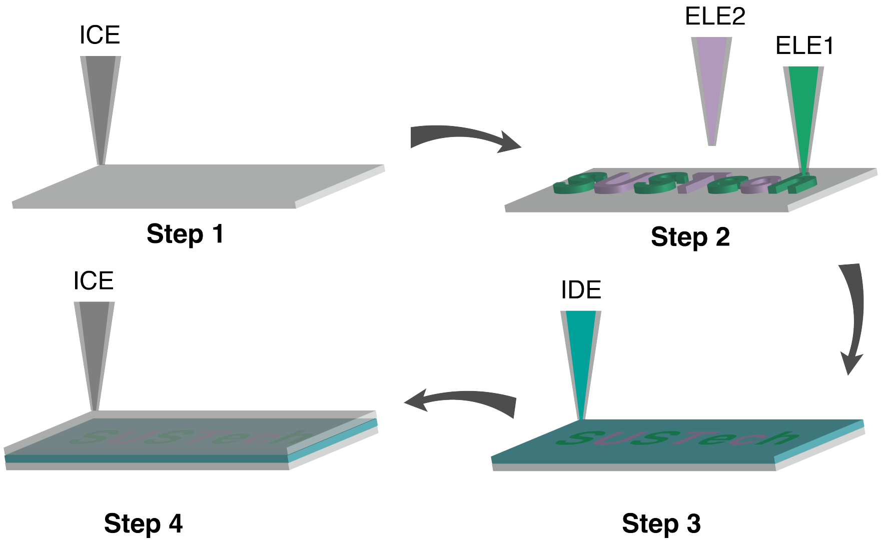

Supplementary Figure 23: **3D printing EL devices through multi-material printing.** Schematic illustrating the 3D printing of a “SUSTech” logo through multi-material printing.

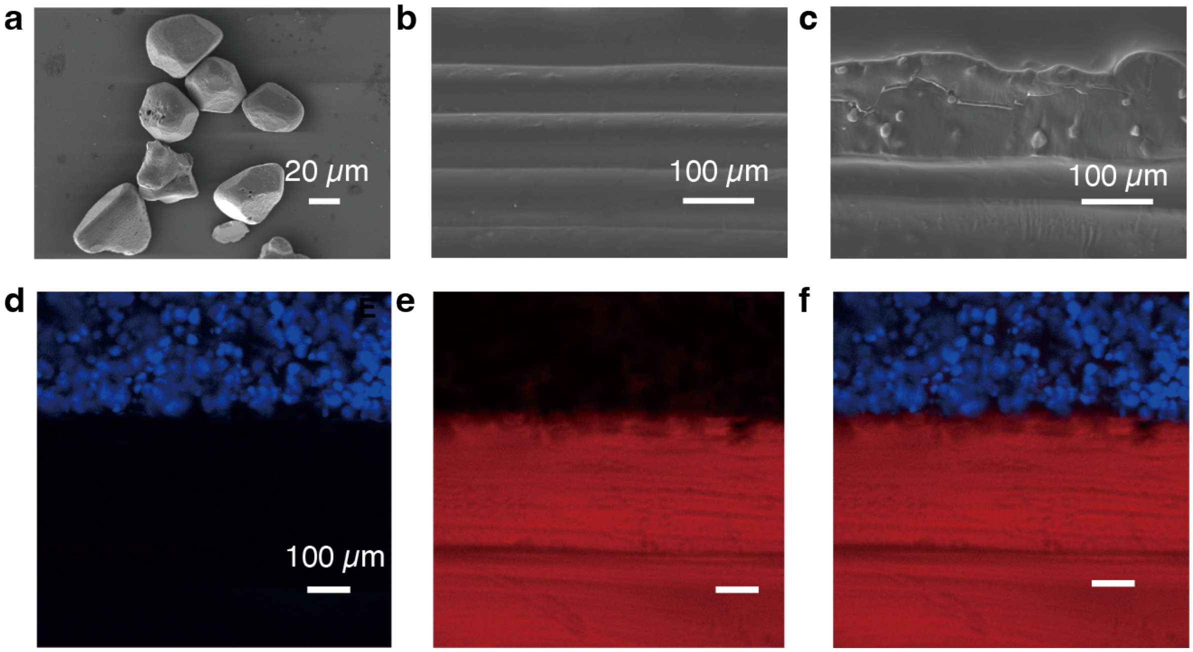

Supplementary Figure 24: **Microscopic morphology of the 3DP ICE/ICE homogeneous interface and the ELE/ICE heterogeneous interface.** **(a)**. SEM image of the electroluminescent ZnS particles with an estimated average particle size of 50 μm. **(b)**. Cross-sectional SEM image of the homogeneous interface between two 3DP ICE layers. **(c)**. Cross-sectional SEM image of the heterogeneous interfaces between a 3DP ICE layer and a 3DP ELE layer. **(d-f)**. Confocal laser scanning microscopic (CLSM) images of the heterogeneous interfaces between a 3DP ELE layer (**d**, the blue luminescence came from the ZnS particles of the ELE layer) and a 3DP ICE layer (**e**, the red luminescence refers to the ICE layer, which was labelled with Nile Red dyes). (**f**) shows the overlapped image of **d** and **e**. Scale bar = 20 μm (**a**) and 100 μm (**b-f**).

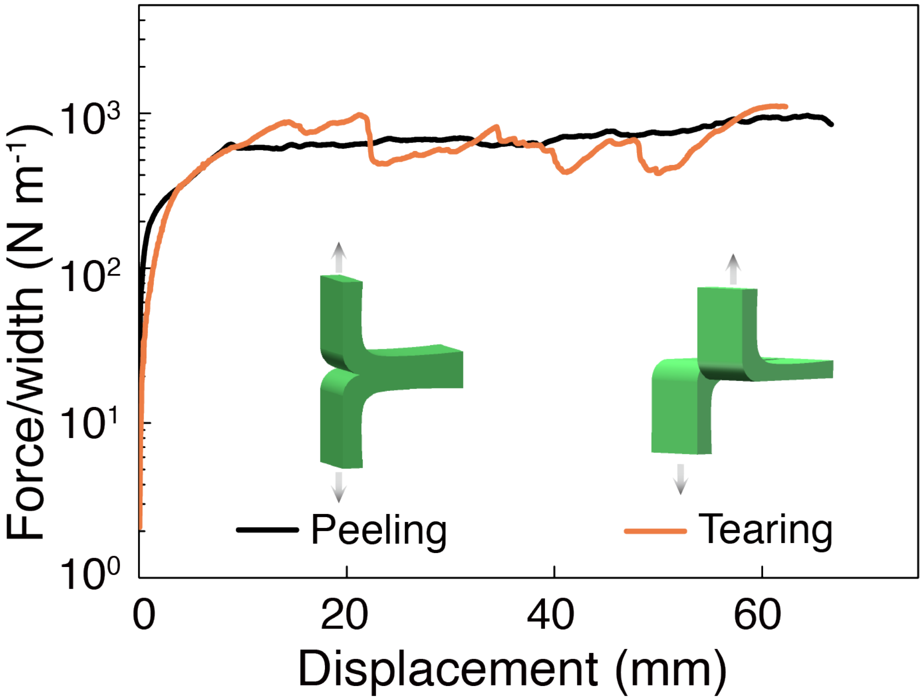

Supplementary Figure 25: **Interfacial toughness of the 3D printed ICE samples.** Representative 180° peeling curves of the 3D printed bilayered ICE samples, while tearing test of the bulk ICE samples was conducted for comparison. Comparable values were obtained, corroborating the robust interface between each layer of the 3DP ICE samples.

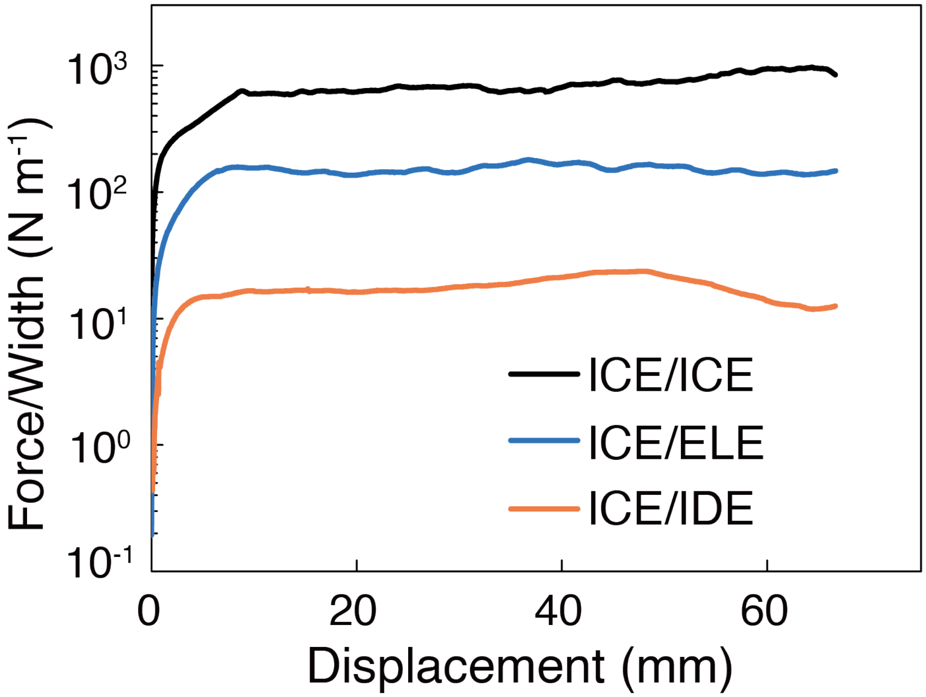

Supplementary Figure 26: **Interfacial toughness of the multi-material 3D printed EL devices.** Representative 180° peeling curves of various interfaces (ICE-ICE, ICE-ELE and ICE-IDE) of the 3D printed EL devices.

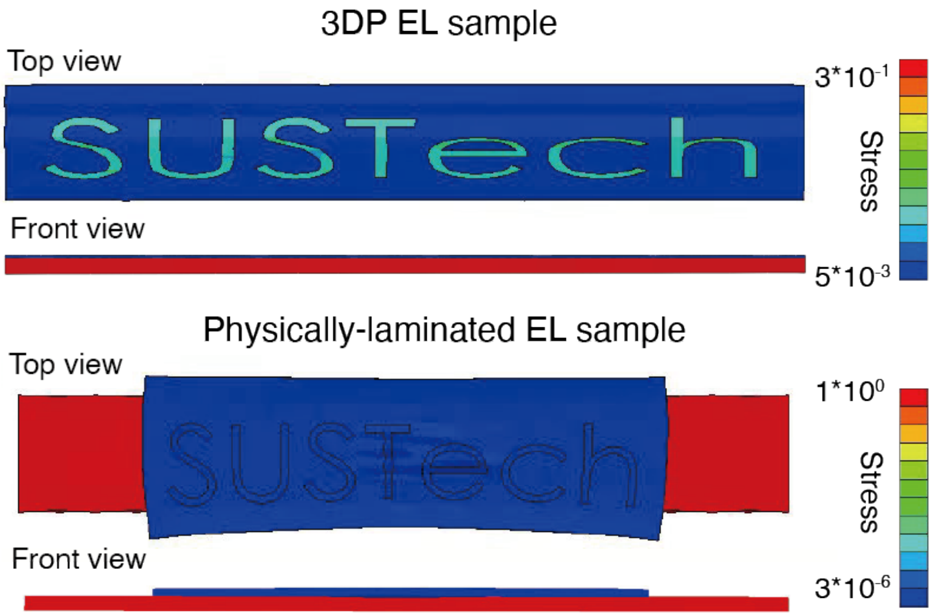

Supplementary Figure 27: **Simulated interfacial stress distribution of the 3D printed EL devices.** Calculated by finite element analysis, the figure shows the simulated stress distributions across the 3DP EL devices (top) and the physically-laminated EL devices (bottom) under an overall applied strain of 35%. The devices consisted of a ELE layer (with "SUSTech" logo) and an ICE layer. The simulation results confirm the mechanically compliant and robust interface of the 3DP EL device, while a weak interface and interface delamination were resulted in the physically-laminated EL device.

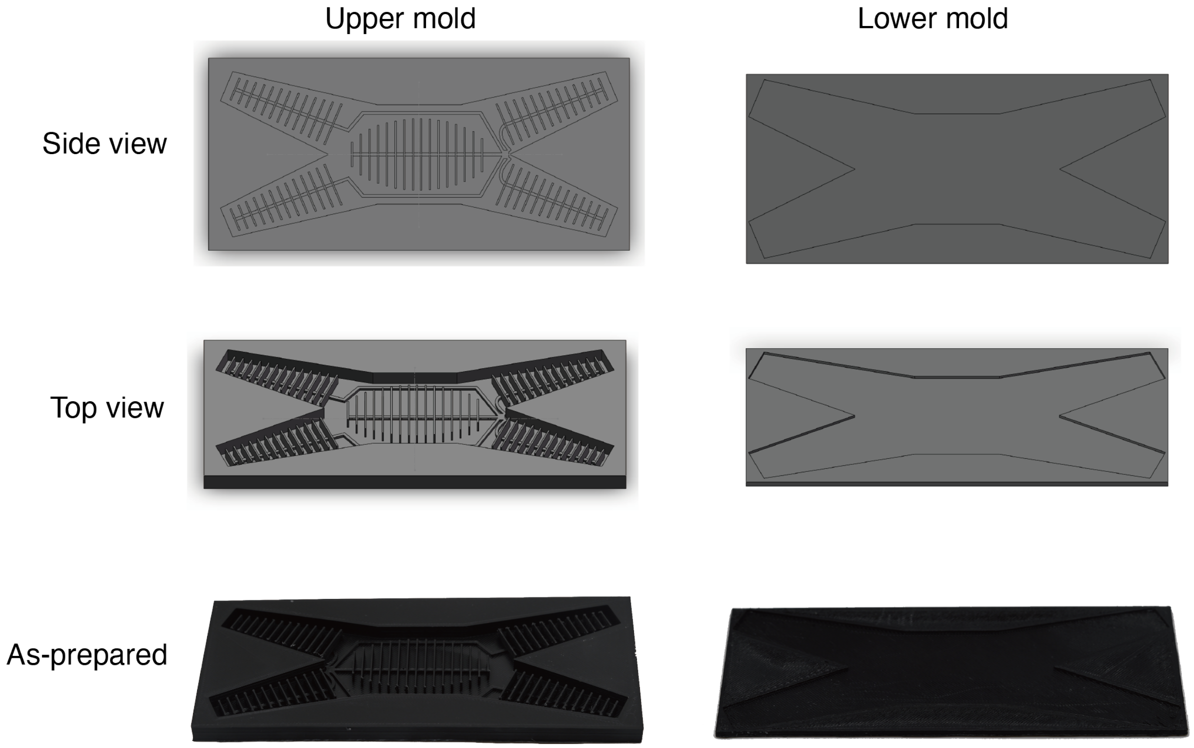

Supplementary Figure 28: **Design of the quadrupedal soft robot.** Schematic illustration of the design of the quadrupedal soft robot from top and side views.

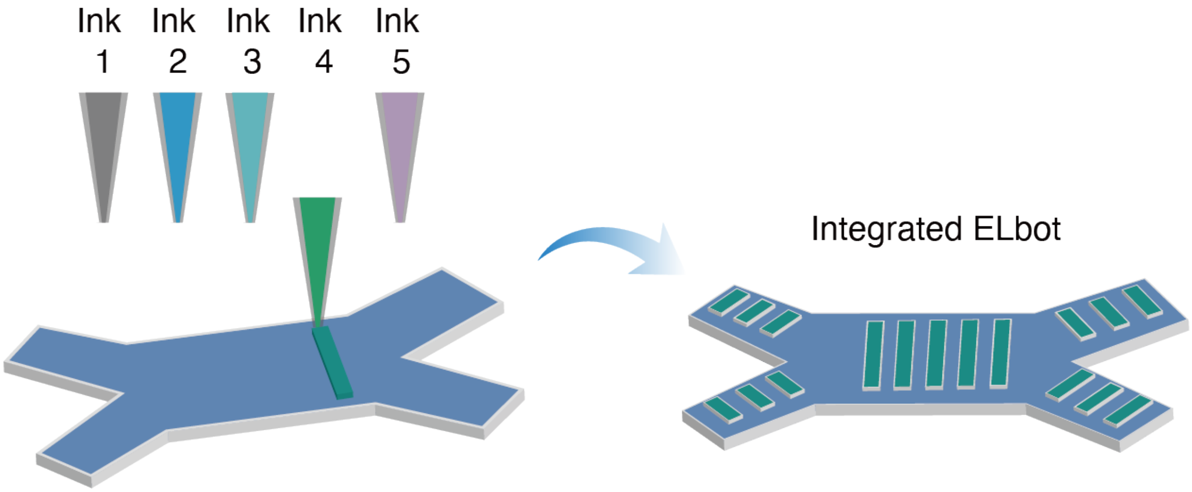

Supplementary Figure 29: **Fabrication of the ELbot.** Schematic illustration showing the printing of the electroluminescent devices on a pneumatic soft robot through multi-material DIW.

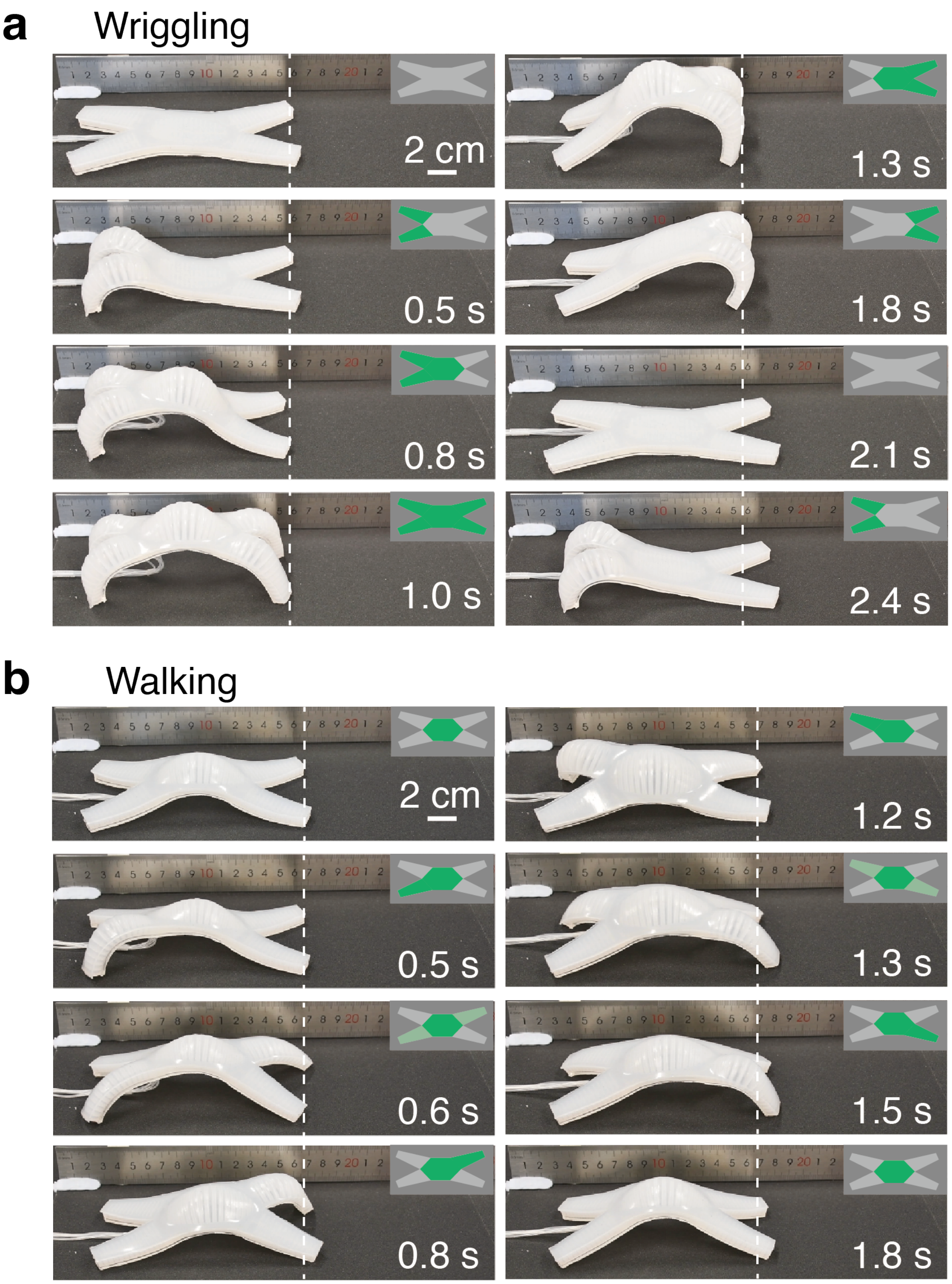

Supplementary Figure 30: **Pneumatically-driven locomotion of the quadrupedal soft robot.** **(a)**. Wriggling motion of the quadrupedal soft robot within 2.4 s. **(b)**. Walking motion of the quadrupedal soft robot within 1.8 s. Inset images illustrate the inflation (as indicated in green) and deflation areas of the five individual channels of the quadrupedal soft robot during the motion.

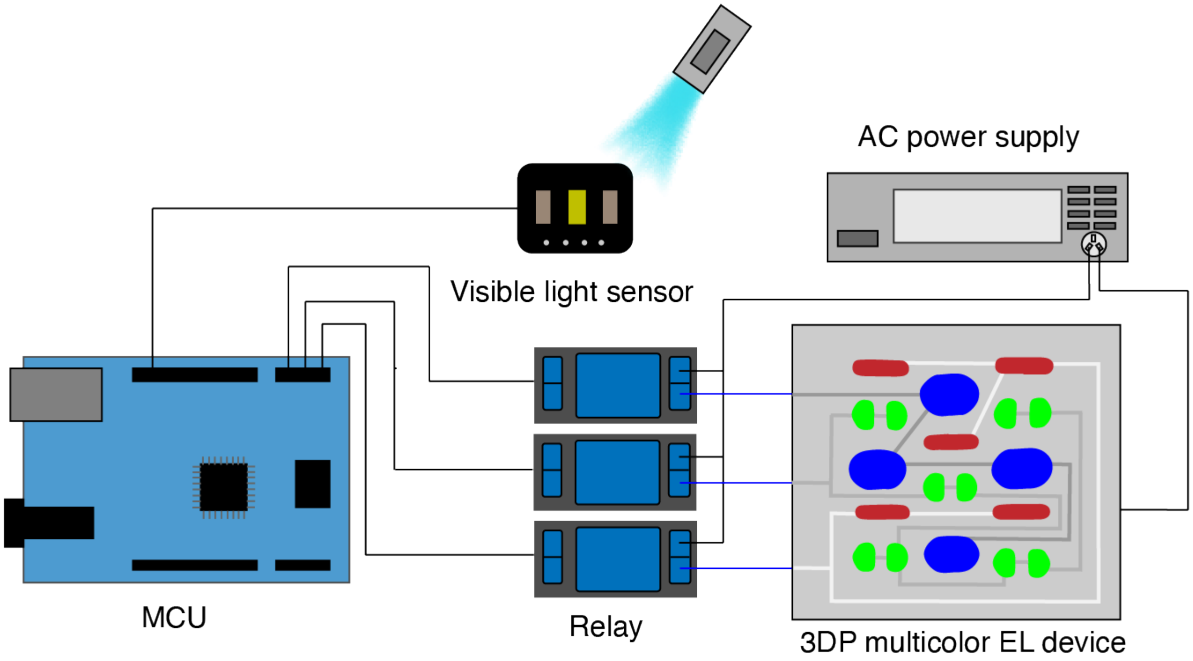

Supplementary Figure 31: **Control logic of the multi-color EL display.** The control system of the multi-color EL display was composed of a microcontroller, a light sensor and three relays, which independently control the AC voltage supplied to the three light-emitting EL devices. The sensor converted the information of the environmental color to electrical information. According to the electrical signal, the microcontroller controlled the ON/OFF of the individual relays, hence the AC voltage supply of the three light-emitting devices.
