# Supplementary material for: Integrated 3D printing of flexible electroluminescent devices and soft robots
Source: Nat Commun. 2022 Aug 23;13:4775. doi: 10.1038/s41467-022-32126-1 (PMC9399151; doi:10.1038/s41467-022-32126-1)
Supplement: Supplementary file 3 — Description of Additional Supplementary Files [file 41467_2022_32126_MOESM3_ESM.pdf]

Description of Additional Supplementary Files

File Name: Supplementary Movie 1
Description: **Mechanical compliance of the 3DP EL device and its robust luminescence under mechanical deformation.** Video showing the flexibility of the 3D printed EL device and its robust electrically-activated luminescence under mechanical deformation, including stretching, bending and twisting. The EL device was operated with an AC power supply of 600 V at 1000 Hz.

File Name: Supplementary Movie 2
Description: **Multi-material 3D printing of a flexible electroluminescent wristband.** Video showing the fabrication process of an electroluminescent wristband through multi-material 3D printing. A “SUSTech" pattern was printed using different light-emitting ELE inks. The bottom and top layers consisted of the ICE ink, and the interlayer consisted of the ELE and IDE inks. The inks were printed through a 210 µm nozzle.

File Name: Supplementary Movie 3
Description: **Crawling motion of the quadrupedal soft robot.** Video showing the pneumatically-driven crawling of a soft robot. The crawling motion was obtained through continuous inflating and deflating of the robot.

File Name: Supplementary Movie 4
Description: **Spatially color-changing ability of the ELbot when crawling from dark to bright environment.** Video demonstrating the adaptability of the ELbot when crawling from dark to bright environment. The EL devices instantly illuminated when the devices were exposed to the bright environmental light. A 200 V AC voltage at a frequency of 1000 Hz was applied.

File Name: Supplementary Movie 5
Description: **Self-adaptive background-matching ability of a 3DP EL display.** Video showing a display with various light-emitting EL units printed on it can emit color-matching light to the environment, when the environmental light was switched to either blue, green or orange.

File Name: Supplementary Movie 6
Description: **Self-adaptive background-matching ability of the ELbot.** Video showing that the 3DP ELbot can simultaneously adapt its appearance to the environmental color when the environmental light was switched to either blue, green or orange. A 200 V AC voltage at a frequency of 1000 Hz was applied.
